# Learning Hierarchical Representations of Stories by Using Multi-Layered Structures in Narrative Multimedia

**DOI:** 10.3390/s20071978

**Published:** 2020-04-01

**Authors:** O-Joun Lee, Jason J. Jung, Jin-Taek Kim

**Affiliations:** 1 Future IT Innovation Laboratory, Pohang University of Science and Technology, Pohang-si 37673, Korea; ojlee112358@postech.ac.kr (O.-J.L.); jintaek@postech.ac.kr (J.-T.K.); 2 Department of Computer Engineering, Chung-Ang University, Seoul 06974, Korea

**Keywords:** hierarchical representation learning, computational narrative analysis, story embedding, Story2Vec, character network

## Abstract

Narrative works (e.g., novels and movies) consist of various utterances (e.g., scenes and episodes) with multi-layered structures. However, the existing studies aimed to embed only stories in a narrative work. By covering other granularity levels, we can easily compare narrative utterances that are coarser (e.g., movie series) or finer (e.g., scenes) than a narrative work. We apply the multi-layered structures on learning hierarchical representations of the narrative utterances. To represent coarser utterances, we consider adjacency and appearance of finer utterances in the coarser ones. For the movies, we suppose a four-layered structure (character roles ∈ characters ∈ scenes ∈ movies) and propose three learning methods bridging the layers: Char2Vec, Scene2Vec, and Hierarchical Story2Vec. Char2Vec represents a character by using dynamic changes in the character’s roles. To find the character roles, we use substructures of character networks (i.e., dynamic social networks of characters). A scene describes an event. Interactions between characters in the scene are designed to describe the event. Scene2Vec learns representations of a scene from interactions between characters in the scene. A story is a series of events. Meanings of the story are affected by order of the events as well as their content. Hierarchical Story2Vec uses sequential order of scenes to represent stories. The proposed model has been evaluated by estimating the similarity between narrative utterances in real movies.

## 1. Introduction

Various studies [1,2,3,4] have been conducted for character networks (i.e., social networks between characters that appear in stories) to analyze stories in narrative multimedia (i.e., creative works that contain stories and are distributed through multimedia) automatically. They applied the analysis results on various applications—summarizing [5,6], recommending [7,8], indexing [9,10], and even generating [11] narrative multimedia. However, the character network model also has limitations. It is not very easy to (i) compare stories by using their character networks and (ii) extract task-agnostic features from character networks.

To resolve these problems, our previous studies [12,13] conducted unsupervised representation learning for structures of the character network. Lee and Jung [12] have proposed three models— Story2Vec-F (Flow-oriented Story2Vec), Story2Vec-D (Denouement-oriented Story2Vec), and Story2Vec-U (Unified Story2Vec). First, they embed snapshots of the social relationships of each character at each moment (Role2Vec). These snapshots are called social roles and used as the smallest unit for the embedding since the relationships represent the roles of characters at the moment. Story2Vec-F and Story2Vec-D embed structures of character networks, based on dynamic changes and compositions of the social roles, respectively. Then, Story2Vec-U is an integration of these two methods. The three models have shown reasonable accuracy for measuring similarity between movie stories. Also, Lee and Jung [13] have proposed a method for representing the content of each scene by learning compositions of social roles in the scene (Scene2Role2Vec).

However, as preliminary studies, they have various limitations. First, stories in narrative multimedia inherently have hierarchical and multi-layered structures (e.g., in the movie, shot ∈ scene ∈ sequence ∈ act ∈ movie). The existing methods cannot consider the structures. Regarding their reasonable accuracy shown in the previous studies [12], this problem may not be severe in representing stories in a single narrative work. However, there are various narrative multimedia distributed as series (e.g., Webtoons [14], Web novels, TV series, etc.), and transmedia storytelling [15] is now an ordinary strategy in the media industry. To deal with these requirements, we need multi-layered representations for stories.

The previous studies [12,13] directly jumped up form the social roles to stories. However, between the social role and the story, there is a significant conceptual gap. The social roles and stories are correlated. However, it is difficult to say the way how they are logically connected. If we climb layers between the social role and story according to hierarchies of utterances on the layers, it will be helpful for the interpretability and explainability of the proposed embedding models.

In this study, we solve those problems by using hierarchical representation learning based on the multi-layered structures of stories. We call utterances on each layer (e.g., social roles, characters, scenes, etc.) ‘narrative utterances.’ Between the social role and the story, we suppose two layers: the character and scene. The character is subjects and objects of events, and the scene is the smallest narrative utterance that describes a concluded event. Therefore, the character and scene appear in the most kinds of narrative multimedia make the proposed model independent form the kinds of media.

There have been a few studies [16,17] for learning hierarchical representations of utterances in natural language documents by extending Word2Vec [18] and Doc2Vec [19]. These studies commonly employed both the distributed memory model of paragraph vector (PV-DM) and the distributed bag of words version of paragraph vector (PV-DBOW) in Doc2Vec to climb the layered structure. PV-DM learns (i) adjacency between finer utterances in coarser utterances. PV-DBOW focuses on only (ii) appearances of the finer utterances in the coarser ones.

We employ this approach and redefine the adjacency and appearance according to narrative utterances on upper and lower layers. From the social role to the character, we focus on that characters have typical roles in a story, and the roles change with the development of the story [20]. A character is represented based on (i) dynamic changes in the social roles of the character and (ii) social roles rooted in character. For the scene, we concentrate on that a scene describes a concluded event. Thus, appearances of characters and relationships among the characters have to be designed to present the event [20,21]. A scene is represented by (i) the existence of relationships between characters in the scene and (ii) characters that appear in the scene. Finally, a story is a series of events, and a scene describes a concluded event [20,21]. The composition and order of scenes in a story can reflect the content and storytelling methods of the story, respectively. Therefore, a story is represented based on (i) temporal adjacency between scenes in the story and (ii) scenes composing the story. The three layers are called as ‘Char2Vec’ (Section 3.1), ‘Scene2Vec’ (Section 3.2), and ‘Hierarchical Story2Vec (Story2Vec-H)’ (Section 3.3), respectively, and this embedding model is called as ‘Hierarchical Story Embedding (HSE).’

To verify the effectiveness of the hierarchical representation learning for the story, we conducted learning representations of characters, scenes, and stories in 142 real movies, as with our previous study [12]. Character vectors, scene vectors, and story vectors were evaluated by measuring the similarity between the characters, between the scenes, and between the stories, respectively. With these experiments, we attempted to validate fundamental assumptions of this study, as follows;

- RQ 1. Multi-layered structures in narrative multimedia are effective for representing narrative utterances on various granularity levels.
- RQ 2. The dynamic changes in social roles of a character are more significant for representing the character than static occurrences of the social roles.
- RQ 3. The character composition in a scene is more effective for representing scenes than social roles appearing in the scene.
- RQ 4. The arrangement of scenes in a story is more useful for representing the story than which social roles appear in the story.

The remainder of this paper is organized as follows. In Section 2, we introduce underlying concepts and definitions of this study, based on the existing story embedding models. We present the proposed method for learning hierarchical representations of narrative utterances, in Section 3. In Section 4, we evaluate the proposed methods by using real movies and validate the fundamental assumptions of this study. Finally, we present the limitations of this study and future research directions in Section 5.

## 2. Background and Preliminaries

This study aims to hierarchically represent narrative utterances (e.g., characters, scenes, and stories) with vectors based on multi-layered structures of stories. Therefore, the HSE model mainly learns relationships between the narrative utterances. Nevertheless, for discovering the relationships, we still rely on the character network. The character network is a social network between characters that appeared in a narrative work. Nodes of the network are characters, edges are relationships between the characters, and weights on the edges are proximity intensity among the characters. The proximity intensity is measured by various methods: the dialogue frequency between characters (directed edges) [3] and the co-occurrence frequency between characters (in-directed edges) [1,22]. This study employs dialogue frequency. Also, we represent the time-sequential characteristics of the story by defining the character network as a dynamic social network. We compose character networks on each scene. The character network is defined as follows:

**Definition** **1** (Character Network [23]). *Suppose that n is the number of characters that appear in a narrative work, $C_{\alpha }$, and $C_{\alpha }$ consists of L scenes from $s_{\alpha ,1}$ to $s_{\alpha ,L}$. When $N(C_{\alpha })$ indicates a character network of $C_{\alpha }$, $N(C_{\alpha })$ can be described as a matrix $\in R^{n\times n}$. Each element of $N(C_{\alpha })$ denotes proximity intensity between two characters. Also, $N(s_{\alpha ,l})$ refers to a character network on the l-th scene $s_{\alpha ,l}$. While $N(C_{\alpha })$ contains interaction among the characters during the entire narrative work, $N(s_{\alpha ,l})$ only includes until $s_{\alpha ,l}$. Thus, character networks on scenes represent a growth process of $N(C_{\alpha })$, and $N\left(s_{\alpha ,L}\right)=N\left(C_{\alpha }\right)$. This can be formulated as:*

(1) $$N\left(C_{\alpha }\right)=N\left(s_{\alpha ,L}\right)=\left[\begin{array}{ccc} a_{1,1} & \cdots & a_{1,n}\\ \vdots & \ddots & \vdots \\ a_{n,1} & \cdots & a_{n,n} \end{array}\right],$$

*where $a_{i,j}$ indicates the proximity of $c_{i}$ to $c_{j}$ when $C_{\alpha }$ is a universal set of characters that appeared in $C_{\alpha }$ and $c_{i}$ is the i-th element of $C_{\alpha }$.*

This study extracts character networks from movie scripts by using CharNet-Extractor (https://github.com/O-JounLee/CharNet-Extractor) developed by Lee et al. [24]. We identify characters by their names and distinguish scenes by scene headings on the scripts. If a character spoke dialogues in a scene, we can recognize the appearance of the character in the scene. Also, we suppose all the characters who appeared in the scene listened to all the dialogues in the scene. When $c_{i}$ and $c_{j}$ appeared on $s_{\alpha ,l}$, and $c_{i}$ spoke a dialogue, we add 1 to $a_{i,j}$ in $N(s_{\alpha ,l})$. Detail methods for composing character networks are presented in our previous study [23].

For embedding character networks, representations of each node or relation are not significant, since characters are unique in a narrative work, as well as other narrative utterances. What we have to represent is the typicality of characters, which can be used for analyzing various stories commonly. Therefore, we first discover substructures in the character network by using the WL (Weisfeiler-Lehman) relabeling process [25]. Based on the substructures, we can represent a story as a set of substructures discovered from its character network.

**Figure 1:**
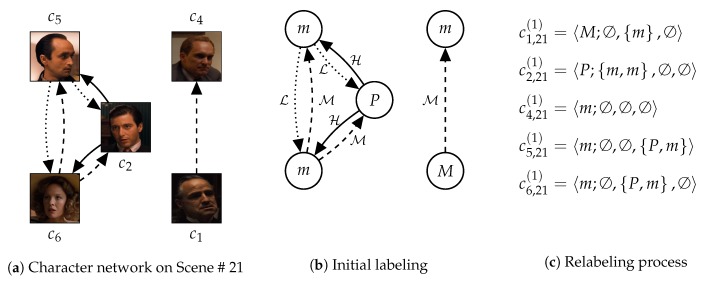
An example of the proximity-aware WL relabeling process on Scene # 21 of ‘The Godfather’ (1972). Nodes indicate characters, and kinds of edges denote proximity intensity between the characters. Indices of the characters are the same as the order of their first appearance. Table 1 presents names and notations of the characters.

The conventional WL relabeling process extracts substructures by only considering adjacency (i.e., the existence of relationships) between characters. However, according to the proximity intensity, relationships between characters can have totally different meanings. Thus, in our previous study [12], we have modified the WL relabeling process to consider proximity between characters. This modification is called as ‘the proximity-aware WL relabeling process’ and described in Figure 1.

As displayed in Figure 1a,b, we first assign initial labels on characters according to their importance. We cluster the characters according to their node centrality into four groups: protagonist, main, minor, and extra characters. Also, to consider proximity, we cluster relationships between characters according to their proximity intensity into three groups: high, medium, and low proximity. These clustering methods have been proposed and verified by the existing studies [1,3,22]. Then, as displayed in Figure 1c, substructures rooted in each character is described by which characters are adjacent to the character with which intensity levels. And, labels of the characters are reassigned according to the descriptions. By repeating (c), the WL relabeling process generates substructures, which have broader coverage and detailed information.

These studies [12,13] call substructures rooted in each character as ‘social roles’ of the character. A social role implicitly represents the character role at a particular scene and scale, despite the absence of the exact meanings. The social role is defined as follows:

**Definition** **2** (Social Role [12]). *Let suppose that $c_{i,l}^{\left(d\right)}$ indicates a social role of $c_{i}$ on $s_{\alpha ,l}$ at degree d∈[0,D]. $c_{i,l}^{\left(d\right)}$ is expressed by one-hop connectivity of $c_{i}$ at d−1 degree. To consider the proximity intensity, this study classifies adjacent characters of $c_{i}$ into the three groups: high (H), medium (M), and low (L) proximity. It is formulated as:*

(2) $$\begin{array}{c} c_{i,l}^{\left(d\right)}=\langle c_{i,l}^{\left(d-1\right)};H{\left(c_{i,l}\right)}^{\left(d\right)},M{\left(c_{i,l}\right)}^{\left(d\right)},L{\left(c_{i,l}\right)}^{\left(d\right)}\rangle , \end{array}$$

*where $H{\left(c_{i,l}\right)}^{\left(d\right)}$, $M{\left(c_{i,l}\right)}^{\left(d\right)}$, and $L{\left(c_{i,l}\right)}^{\left(d\right)}$ indicate sets of social roles rooted in neighborhoods of $c_{i}$ at d−1 degree. These sets include neighborhoods that receive high, medium, and low proximity from $c_{i}$, respectively.*

The social role enables us to represent a story as a multi-set of social roles, like a document as a multi-set of words. Thereby, we can embed social roles and stories based on appearances of the social roles in the stories, by extending Word2Vec [18] and Doc2Vec [19]. However, fixed-size windows of Word2Vec and Doc2Vec are not adequate for graphical data, since the number of adjacent nodes in networks is not constant. Thus, our previous studies [12,13] employed radial neighborhoods in Subgraph2Vec [26] and Graph2Vec [27], which define neighboring substructures based on (i) adjacency of nodes (e.g., $c_{i,l}^{\left(d\right)}$ and $c_{j,l}^{\left(d\right)}$ on Figure 1) and (ii) adjacency of degrees (e.g., $c_{i,l}^{\left(d\right)}$, $c_{i,l}^{\left(d-1\right)}$, and $c_{i,l}^{\left(d+1\right)}$). Our previous studies extended the radial neighborhoods to the temporal-radial neighborhoods by also applying (iii) adjacency of scenes (e.g., $c_{i,l}^{\left(d\right)}$, $c_{i,l-1}^{\left(d\right)}$, and $c_{i,l+1}^{\left(d\right)}$).

By extending the range of neighborhoods in SkipGram of Word2Vec [18], we have been proposed Role2Vec for representing social roles with a vector [12]. For a social role, Role2Vec maximizes its co-occurrence probability with its neighboring social roles and minimizes the probability with the other social roles. This is formulated as:

(3) $$\begin{array}{cc} L_{S}\left(c_{i,l}^{\left(d\right)}\right)= & \sum_{\forall S_{a}\in N^{F}\left(c_{i,l}^{\left(d\right)}\right)}\log P\left(S_{a}|\Phi \left(c_{i,l}^{\left(d\right)}\right)\right)-\sum_{\forall S_{b}\notin N^{F}\left(c_{i,l}^{\left(d\right)}\right)}\log P\left(S_{b}|\Phi \left(c_{i,l}^{\left(d\right)}\right)\right), \end{array}$$

where $\Phi \left(\cdot \right)$ indicates the projection function, $S_{a}$ denotes the *a*-th social role, and $N^{F}\left(\cdot \right)$ indicates a set of social roles that are in temporal-radial neighborhoods.

Then, based on compositions of social roles in stories, we have proposed Story2Vec models for representing stories with a vector [12]. First, Story2Vec-F embeds dynamic changes in structures of character networks by extending PV-DM of Doc2Vec [19]. For a social role in a story, Story2Vec-F maximizes its co-occurrence probability with its neighboring social roles and the story and minimizes the probability with the other social roles. This can be formulated as:

(4) $$\begin{array}{cc} L_{F}\left(C_{\alpha }\right)= & \sum_{\begin{array}{c} 0\leq d\leq D,\\ 1\leq l\leq L,\\ c_{i}\in C_{\alpha } \end{array}}\left[\log P\left(c_{i,l}^{\left(d\right)}|N^{F}\left(c_{i,l}^{\left(d\right)}\right),\Phi \left(C_{\alpha }\right)\right)-\sum_{\forall S_{b}\neq c_{i,l}^{\left(d\right)}}\log P\left(S_{b}|N^{F}\left(c_{i,l}^{\left(d\right)}\right),\Phi \left(C_{\alpha }\right)\right)\right]. \end{array}$$

Story2Vec-D embeds static structures of character networks (only on the last scene) by extending PV-DBOW of Doc2Vec [19]. For a story, Story2Vec-D maximizes an occurrence probability of social roles appearing in the story and minimizes the probability of the other social roles. This is formulated as:

(5) $$\begin{array}{cc} L_{D}\left(C_{\alpha }\right)= & \sum_{\begin{array}{c} 0\leq d\leq D,\\ c_{i}\in C_{\alpha } \end{array}}\left[\log P\left(c_{i,L}^{\left(d\right)}|\Phi \left(C_{\alpha }\right)\right)-\sum_{\forall S_{b}\notin S\left(s_{\alpha ,L}\right)}\log P\left(S_{b}|\Phi \left(C_{\alpha }\right)\right)\right], \end{array}$$

where $S\left(s_{\alpha ,L}\right)$ indicates a set of social roles discovered from $N\left(s_{\alpha ,L}\right)$. Finally, Story2Vec-U embeds both of dynamic and static structures of character networks by integrating Story2Vec-F and Story2Vec-D. An objective function of Story2Vec-U is a summation of the objective functions of Story2Vec-F and Story2Vec-D, as $L_{U}\left(C_{\alpha }\right)=L_{F}\left(C_{\alpha }\right)+L_{D}\left(C_{\alpha }\right)$.

However, the three models suffered from (i) the conceptual gap between social roles and stories and (ii) the excessive diversity of social roles. If we add layers between the social roles and stories, the burdens will also be distributed to the additional layers. Thus, we propose the hierarchical embedding model for the story, which can easily accommodate various narrative utterances. For learning methods on each layer, we employ both PV-DM and PV-DBOW of Doc2Vec [19]. Also, for the hierarchical learning, we define an objective function of the entire model as a summation of objective functions for every narrative utterance on all the layers. PV-DM and PV-DBOW learn representations based on the adjacency and appearance of smaller utterances (on a lower layer) in bigger utterances (on an upper layer). Therefore, to add and connect layers, we have to define narrative utterances on each layer, adjacency between the utterances on each layer, and inclusion of the utterances between adjacent layers.

In this study, we add characters and scenes. Although there are various kinds of narrative utterances, they do not commonly appear in diverse narrative multimedia. For example, ‘sequences’ are only in visual narratives, and we can use ‘episodes’ only for serials, such as TV series and webtoons. Also, in the visual narratives, we still do not have methods for detecting boundaries of sequences or acts with enough accuracy, at the extent of our knowledge [5,12]. To add layers, we first clarify the relationships between social roles, characters, scenes, and stories. We narrow down the temporal-radial neighborhoods into a character as follows:

**Definition** **3** (Social Role-Character Relationship). *A Character has multiple social roles on each scene and degree. Social roles on adjacent scenes show temporal changes in the character. On the other hand, social roles on adjacent degrees exhibit various meanings of the character according to the observation range. Thus, adjacency between social roles is determined by roles on the adjacent scenes and degrees. Also, appearance of the social roles is decided by which social roles are rooted in the character.*

The character network is a representation of relationships between characters and scenes as follows:

**Definition** **4** (Character-Scene Relationship). *If a character $c_{i}$ appears on $s_{\alpha ,l}$, $c_{i}$ has a role in an event described by $s_{\alpha ,l}$. $N(s_{\alpha ,l})$ represents relationships between characters appeared on $s_{\alpha ,l}$. A component $a_{i,j}$ of $N(s_{\alpha ,l})$ signifies frequency of interactions that have $c_{i}$ as a subject and $c_{j}$ as an object. Thus, when $a_{i,j}\neq 0$ or $a_{j,i}\neq 0$, $c_{i}$ and $c_{j}$ are adjacent in $N(s_{\alpha ,l})$. Also, orthogonal components of $N(s_{\alpha ,l})$ (e.g., $a_{i,i}$ for $c_{i}$) indicates appearance of characters.*

In most of the narrative work, scenes are sequentially presented as follows:

**Definition** **5** (Scene-Story Relationship). *A narrative work can be represented as a sequence of scenes: $C_{\alpha }=\langle s_{\alpha ,1},\cdots ,s_{\alpha ,l},\cdots ,s_{\alpha ,L}\rangle$. Also, $N(C_{\alpha })$ and $N(s_{\alpha ,l})$ reflect content of the narrative work and scene. Therefore, order of scenes determines their adjacency; for example, $s_{\alpha ,l-1}$, $s_{\alpha ,l}$, and $s_{\alpha ,l+1}$. Obviously, components of $\langle s_{\alpha ,1},\cdots ,s_{\alpha ,l},\cdots ,s_{\alpha ,L}\rangle$ decide appearance of the scenes in $C_{\alpha }$.*

There have been various studies for learning representations of networks and entities in the networks (e.g., nodes, edges, substructures, etc.) [28,29,30]. Also, there have been various network embedding methods [31,32,33,34] that exhibited the state-of-the-art performance. However, our story embedding studies [12,13] keep applying the Word2Vec-based methods [26,27]. There are two main reasons. First, the existing methods mostly aim at social networks [31,34] or knowledge graphs [35,36]. They mainly attempted to embed nodes or edges for the link prediction, community detection, and so forth. Nevertheless, although we also embed characters, which are nodes of character networks, the other narrative utterances that we aim to have barely got attention from the existing studies. For example, a story corresponds to a dynamic network, a scene can be said as a snapshot of the dynamic network, and a social role is a snapshot of a node in the network. Even, we attempt to represent typicality of the character, not just each character. Thus, we have to deal with the character as a node of multiple dynamic networks. The Word2Vec-based models are relatively easy to embrace the diversity of narrative utterances due to the simplicity of their approach. What we only need is clear and precise definitions for the narrative utterances and relationships between the utterances (e.g., adjacency and appearance).

Second, simply speaking, we need a method for multi-resolution embedding on multi-dynamic networks. Du and Tong [37] have proposed a method for multi-resolution multi-network embedding, which is based on the WL relabeling and Word2Vec (SkipGram and negative sampling). Also, their method exhibited the state-of-the-art performance. We anticipate that this approach will show reliable performance on learning hierarchical representations of narrative utterances based on character networks.

## 3. Learning Hierarchical Representations of Narrative Utterances

**Figure 2:**
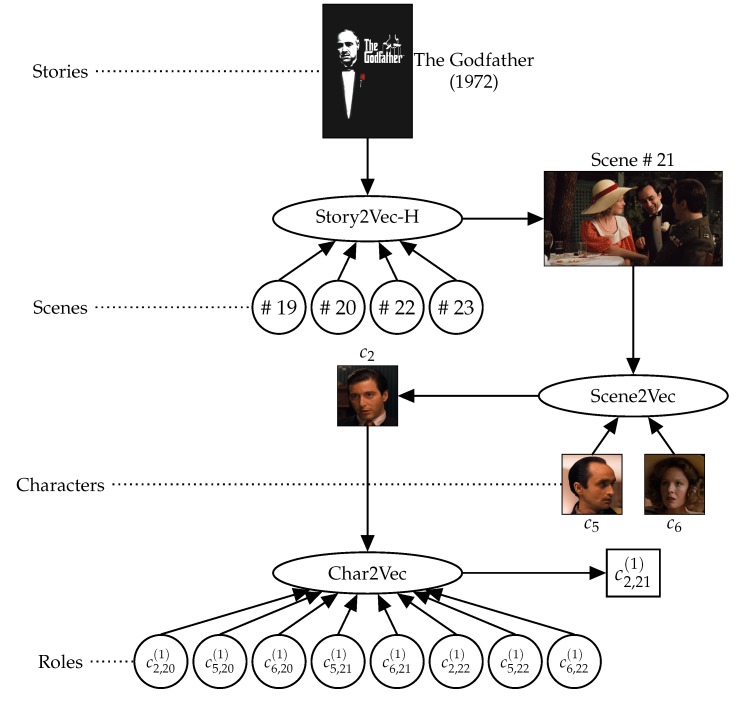
An example of the hierarchical story embedding (HSE) model for ‘The Godfather’ (1972). This study presents the learning procedures of the HSE model that consists of four layers: the social role, character, scene, and story. Elliptical nodes indicate learning methods that bridge the adjacent layers. Nodes, which are beside the leaning methods, denote prediction targets of the methods. Nodes below the learning methods refer to narrative utterances on each layer. Also, they are neighborhoods of the target utterances (context) for the distributed memory model of paragraph vector (PV-DM) parts; the distributed bag of words version of paragraph vector (PV-DBOW) parts learn appearances of all the utterances on the lower layers.

We propose a model for learning representations of various narrative utterances hierarchically, as described in Figure 2. As with the existing studies [16,17], we employ both PV-DM and PV-DBOW methods of Doc2Vec [19] on each layer (i.e., granularity of narrative utterances). To train representation of an utterance on the i+1-th layer, PV-DM and PV-DBOW learn adjacency and appearance of utterances on the *i*-th layer, respectively. Narrative utterances are diverse and different according to kinds of media. Thus, this study adds only the character and scene layers between the social role and story, since they commonly appear in all the narrative multimedia. In the following sections, we introduce the methods how we can climb the layers based on Definitions 3–5.

### 3.1. Representing Characters Based on Dynamic Changes in Social Roles

On all the layers, the HSE model applies PV-DM and PV-DBOW approaches together. A difference between the layers is definitions for the adjacency and appearance of narrative utterances on each layer. According to Definition 3, Char2Vec determines the appearance of social roles in terms of characters that the social roles rooted in. For a character $c_{i}$, we consider all the social roles of $c_{i}$ that appeared from $s_{\alpha ,1}$ to $s_{\alpha ,L}$ at d=1 to *D* degrees. Adjacency between social roles is decided by fixed-length windows: $W_{T}$ for narrative time and $W_{D}$ for scale. Neighborhoods of a social role rooted in $c_{i}$ at $s_{\alpha ,l}$ on *d* degree consist of social roles of $c_{i}$ on scenes from $s_{\alpha ,l-W_{T}}$ to $s_{\alpha ,l+W_{T}}$ at $d-W_{D}$ to $d+W_{D}$ degrees. This is called as ‘temporal neighborhoods’ and formulated as:

(6) $$\begin{array}{cc} N^{T}\left(c_{i,l}^{\left(d\right)}\right)= & \{c_{i,l+\Delta L}^{\left(d+\Delta D\right)}|\left|\Delta D\right|\leq W_{D},\left|\Delta L\right|\leq W_{T},c_{i,l+\Delta L}^{\left(d+\Delta D\right)}\neq c_{i,l}^{\left(d\right)}\}. \end{array}$$

This range is also illustrated by the lower part of Figure 2.

By using the well-known movie ‘The Godfather’ (1972), Figure 3 presents an example of relationships between the adjacency of social roles, characters, and scenes. With this example, we show empirical evidences supporting our definitions for the relationships (Definitions 3–5). In this section, we mainly discuss the adjacency of social roles in characters. Additionally, Table 1 presents a list of the major characters that appear in ‘The Godfather’ (1972).

**Figure 3:**
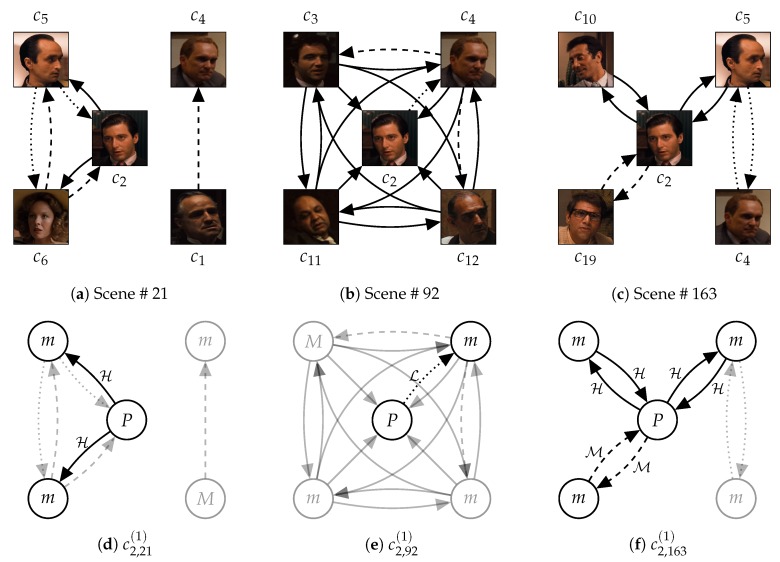
Examples of relationships between social roles, characters, scenes, and stories. (**a**–**c**) represent the social relationships between characters on Scene # 21, # 92, and # 163 in ‘The Godfather’ (1972), respectively. Then, (**d**–**f**) describe social roles of ‘Michael Corleone’ ($c_{2}$) at degree d=1 on Scene # 21, # 92, and # 163, respectively. From (**d**) to (**f**), we can see the meanings of time-sequential changes in social roles. (**e**) shows the significance of adjacent degrees of social roles. (**a**–**c**) show the way how relationships of characters are designed to convey the content of the scenes. Finally, also based on (**a**–**c**), we can find out the meaning of the order of scenes.

Figure 3d–f present the reason why time-sequential changes in social roles are meaningful for representing characters and stories. Scene # 21 alternately described the wedding ceremony of ‘Connie’ and the business meeting of the mafia family. On the scene, ‘Michael Corleone’ ($c_{2}$) interacted only with ‘Fredo’ ($c_{5}$) and ‘Kay Adams’ ($c_{6}$) who were far from the family business. Scene # 92 described a family meeting after an assassination attempt for ‘Don Vito Corleone’ ($c_{1}$) and before the revenge of ‘Michael’ for the assassination. Although ‘Michael’ participated in the family meeting on Scene # 92, he only interacted with ‘Tom Hagen’ ($c_{4}$). However, the interaction frequency was still low, and he did not react to conversations between the other family members. Finally, on Scene # 163, ‘Michael’ became a mafia boss by succeeding his father. On the scene, he showed high proximity to his family members. This example shows conflicts, inner sides, and personalities of characters are reflected by their social roles. Also, dynamic changes in social roles represent the typicality of ‘Michael,’ who gave up their own belief for their family (important persons).

Figure 3e illustrates that social roles at each degree can be interpreted differently according to their scales. The social role of ‘Michael’ ($c_{2}$) on Scene # 92 at degree 1 only represents that he has low proximity to ‘Tom’ ($c_{4}$). At degree 2, we can acquire information that ‘Tom’ was interacting with ‘Sonny Corleone’ ($c_{3}$) and ‘Tessio’ ($c_{12}$) at the same time. As the degree getting deeper, we can find out that Scene # 92 described the meeting between mafia members. Finally, the social roles of ‘Michael’ on Scene # 92 indicate that he started participating in the family meeting, but still passive.

Char2Vec represents characters with a vector based on the changes in their social roles according to the narrative time and observation range. The PV-DM part learns the continuous changes at each moment and scale, and the PV-DBOW part learns the static overview of the changes. However, in our previous study [12], Story2Vec-F (based on PV-DM) suffered from that appearance of each social role was diluted while tracing a massive amount of the changes. The PV-DBOW part will be able to complement this problem by emphasizing snapshots of a character on each scene.

The PV-DM part estimates a co-occurrence probability of a social role with its neighborhoods in a character that contains the social role. We integrate these multiple conditions (neighborhoods and the target character) into a single vector by averaging vector representations of the adjacent social roles and the character. Our objective function maximizes the co-occurrence probability between adjacent social roles within the target character and minimizes the probability between nonadjacent social roles. Then, the loss for a character is calculated by a summation of losses for all social roles rooted in the character. This can be formulated as:

(7) $$\begin{array}{cc} L_{\mathrm{CA}}\left(c_{i}\right)= & \sum_{\begin{array}{c} 0\leq d\leq D\\ 1\leq l\leq L \end{array}}\left[\log P\left(c_{i,l}^{\left(d\right)}|N^{T}\left(c_{i,l}^{\left(d\right)}\right),\Phi \left(c_{i}\right)\right)-\sum_{S_{b}\neq c_{i,l}^{\left(d\right)}}\log P\left(S_{b}|N^{T}\left(c_{i,l}^{\left(d\right)}\right),\Phi \left(c_{i}\right)\right)\right]\\ ≃ & \sum_{\begin{array}{c} 0\leq d\leq D\\ 1\leq l\leq L \end{array}}\left[\log\sigma \left(\Phi {\left(c_{i,l}^{\left(d\right)}\right)}^{⊺}\Phi_{C}\left(c_{i,l}^{\left(d\right)}\right)\right)+\sum_{j=1}^{k}E_{S_{b}∼P_{n}\left(S\right)}\left[\log\sigma \left(-\Phi {\left(S_{b}\right)}^{⊺}\Phi_{C}\left(c_{i,l}^{\left(d\right)}\right)\right)\right]\right], \end{array}$$

where $\Phi_{C}\left(\cdot \right)$ denotes a vector representation for the context (conditions), σ(·) indicates the sigmoid function, $P_{n}\left(S\right)\propto U{\left(S\right)}^{w}$ denotes a noise distribution of social roles, and $U\left(S\right)$ refers to a unigram distribution of social roles.

Narrative works contain various characters, and the social roles of the characters are barely duplicated. Thus, we have to deal with relatively numerous social roles, as compared with the number of narrative works. For example, our movie corpus includes 142 movies and 37,631 social roles. Therefore, we sample the nonadjacent social roles according to the noise distribution ($P_{n}\left(S\right)$) by using the negative sampling. This method enables us to estimate the expectation of losses for the nonadjacent social roles. When w∈[0,1] is close to 0, negative samples are randomly sampled; otherwise, according to the occurrence probability. The noise distribution is also used for Role2Vec, and we empirically tuned *w*. The number of negative samples (*k*) is determined as the average size of the neighborhoods, as with our previous study [12]. Additionally, role vectors (i.e., vector representations for social roles) are pre-trained by the Role2Vec method [12], and character vectors are initially set by averaging role vectors of social roles rooted in corresponding characters.

All the PV-DBOW parts in the HSE model and Story2Vec-D share the common learning strategy, and the only difference between them is their definitions of appearance. An objective function of the PV-DBOW part is defined by combining the negative sampling and PV-DBOW, as with Equation (5). For a character $c_{i}$, we maximize an occurrence probability of every social role rooted in $c_{i}$ and minimize the probability for social roles that are not rooted in $c_{i}$. This is formulated as:

(8) $$\begin{array}{cc} L_{\mathrm{CO}}\left(c_{i}\right)= & \sum_{\begin{array}{c} 0\leq d\leq D\\ 1\leq l\leq L \end{array}}\left[\log P\left(c_{i,l}^{\left(d\right)}|\Phi \left(c_{i}\right)\right)-\sum_{\forall S_{b}\notin S\left(c_{i}\right)}\log P\left(S_{b}|\Phi \left(c_{i}\right)\right)\right]\\ ≃ & \sum_{\begin{array}{c} 0\leq d\leq D\\ 1\leq l\leq L \end{array}}\left[\log\sigma \left(\Phi {\left(c_{i,l}^{\left(d\right)}\right)}^{⊺}\Phi \left(c_{i}\right)\right)+\sum_{j=1}^{k}E_{S_{b}∼P_{n}\left(S\right)}\left[\log\sigma \left(-\Phi {\left(S_{b}\right)}^{⊺}\Phi \left(c_{i}\right)\right)\right]\right], \end{array}$$

where $S\left(c_{i}\right)$ denotes a set of social roles rooted in $c_{i}$. To integrate the PV-DM and PV-DBOW parts, we define an objective function of Char2Vec as a summation of the objective functions of the two parts, as $L_{C}\left(c_{i}\right)=L_{\mathrm{CA}}\left(c_{i}\right)+L_{\mathrm{CO}}\left(c_{i}\right)$.

**Figure 4:**
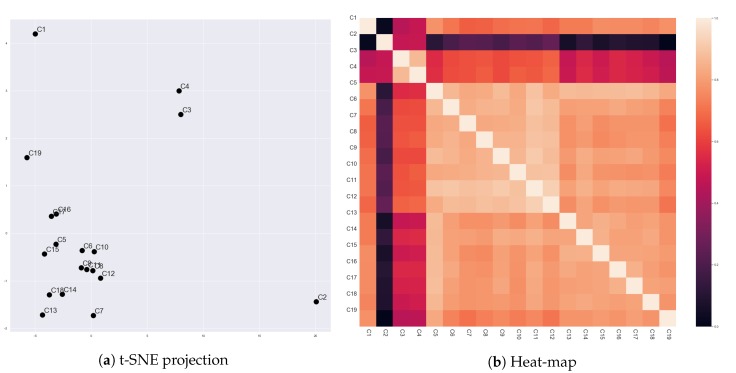
Vector representations of characters in ‘The Godfather’ (1972) that were generated by Char2Vec. Dots on (**a**) denote 19 characters that appeared in ‘The Godfather’ (1972), excluding extras. Since the vector representations (which are 32-dimensional) have been projected into 2-dimensions using t-SNE, the axes of (**a**) are not significant. (**b**) presents a heat-map for similarity between the character vectors. The similarity was calculated by Equation (16).

Figure 4 presents vector representations of characters in ‘The Godfather’ (1972) that were generated by Char2Vec. Since our movie corpus, which consists of 142 movies, includes numerous characters, we only use characters in ‘The Godfather’ (1972) for discussion. A comparison between their character vectors can show whether the character vectors reflect changes in the social relationships of characters. In ‘The Godfather’ (1972), only ‘Don Vito Corleone’ ($c_{1}$) and ‘Michael Corleone’ ($c_{2}$) exhibit significant changes. Before the assassination attempt for ‘Vito Corleone’, ‘Michael’ showed a passive stance for his family members, and ‘Vito’ actively lead the Corleone family. After the assassination, ‘Michael’ started participating in the family business and became the mafia boss finally. On the other hand, ‘Vito’ lost his energy to manage the family business. Both ‘Vito’ and ‘Michael’ significantly change but in opposite directions. Thus, locations of their vector representations were not only far from other characters, but also far from each other.

Excluding ‘Vito’ and ‘Michael,’ the other characters show relatively constant social relationships. Among these characters, ‘Sonny Corleone’ ($c_{3}$) and ‘Tom Hagen’ ($c_{4}$) showed changes. However, the changes were not as significant as ‘Vito’ and ‘Michael’. ‘Sonny Corleone’ and ‘Tom Hagen’ were constantly hot-blooded and cautious, respectively. After the assassination attempt for ‘Vito’, ‘Sonny’ started leading the Corleone family. He changed more active than under the control of ‘Vito’ and insisted radical reactions for the attempt. Due to the rampancy of ‘Sonny’, ‘Tom’ started having conflicts with him. Thus, ‘Tom’ also changed more active than when ‘Vito’ leads the family. However, these changes were not as significant as making changes in their personalities. Vector representations for ‘Sonny’ and ‘Tom’ reflected these points. They were distant from the other constant characters, but also far from the two dynamic characters. At the same time, ‘Sonny’ and ‘Tom’ were relatively close to each other. This result might come from that their directions of changes were similar. Additionally, Fredo ($c_{5}$) kept timid, Clemenza ($c_{11}$) was constantly royal, and Tessio ($c_{12}$) betrayed the Corleone family. Nevertheless, their differences were not significantly represented since they have low interaction frequency commonly.

These results exhibited that Char2Vec could distinguish round characters from flat characters. The round and flat characters indicate characters that have dynamic changes in their personalities and do not have, respectively [38]. Stories are lead by conflicts around protagonists and a few main characters, and the conflicts force the characters to be changed [20]. Therefore, the results also say that Char2Vec can find which characters are leading the stories.

### 3.2. Representing Scenes based on Character Occurrences

To represent each scene with a vector, this study assumes that an event in a story corresponds to a scene. Thus, the characters and their interactions on the scene are composed to describe the event. Figure 3a–c present interactions between characters on the three scenes (Scene # 21, # 92, and # 163) in ‘The Godfather’ (1972). Scene # 21 contrasted characters that were not involved in the mafia business with other characters who were the mafia members. In the same background (the wedding ceremony), the two groups of characters were separate. On Scene # 92, the Corleone family had a meeting to discuss the revenge for assassination conspiracy against ‘Don Vito Corleone’ ($c_{1}$). Thus, all the key members of the Corleone family appeared on the scene. The passive attitude of ‘Michael Corleone’ ($c_{2}$) and ‘Tom Hagen’ ($c_{4}$) represented their positions (a civilian and a lawyer) that they were not fully assimilated in mafia members. Scene # 163 described another business meeting of the mafia family. The composition of characters on this scene is similar to the business meetings hosted by ‘Vito’ at the beginning part of the movie. ‘Michael’ replaced ‘Vito,’ ‘Sonny Corleone’ ($c_{3}$) disappeared, but ‘Tom’ was still there. This scene said that ‘Michael’ finally succeeded his father (‘Vito’). Obviously, without the content of the movie, we can not perfectly interpret the meanings of events described in each scene. Nevertheless, this example shows us that the social relationships between characters reflect the meanings of events, at least partially.

To learn relationships among characters, the adjacency between characters is determined by their interaction frequency (proximity) on each scene, as described in the middle part of Figure 2. If a character $c_{i}$ has an interaction with another character $c_{j}$ on a scene $s_{\alpha ,l}$, a proximity degree of $c_{i}$ for $c_{j}$ ($a_{i,j}$) on a character network of $s_{\alpha ,l}$ ($N(s_{\alpha ,l})$) is not 0. This can be formulated as:

(9) $$\begin{array}{cc} N^{R}\left(c_{i},s_{\alpha ,l}\right)= & \{c_{j}\;|\;N\left(s_{\alpha ,l}\right)∋a_{i,j}\neq 0\}. \end{array}$$

Different from the existing methods (Role2Vec and Story2Vec-F/D/U) and Char2Vec, Scene2Vec does not employ the negative sampling due to the following reasons. First, since characters are mostly unique utterances of each narrative work, it is difficult to define a noise distribution of characters (it will be a uniform distribution). Second, in most of the movies (which is the experimental subject), the number of characters is no more than two dozens.

Therefore, in the PV-DM part, a co-occurrence probability of a character on a target scene is estimated based on its neighboring characters and the scene. Since we do not use the negative sampling, the co-occurrence probability is calculated by the softmax function instead of the sigmoid function. In here, all the negative samples are reflected in a denominator of the softmax function without sampling processes. As with Char2Vec, a representative vector of the context is made by averaging vector representations of a scene and characters in the context. Thereby, its objective function is designed to maximize the co-occurrence probability of characters that have social relationships with a target character and minimize the probability for characters that do not interact with the target character on the scene. This is formulated as:

(10) $$\begin{array}{cc} L_{\mathrm{SA}}\left(s_{\alpha ,l}\right)= & \sum_{\begin{array}{c} \forall c_{i}\in C_{\alpha ,l} \end{array}}\log P\left(c_{i}|N^{R}\left(c_{i},s_{\alpha ,l}\right),\Phi \left(s_{\alpha ,l}\right)\right)≃\sum_{\begin{array}{c} \forall c_{i}\in C_{\alpha ,l} \end{array}}\frac{exp\left(\Phi {\left(c_{i}\right)}^{⊺}\Phi_{R}\left(c_{i}\right)\right)}{\sum_{\begin{array}{c} \forall c_{j} \end{array}}exp\left(\Phi {\left(c_{j}\right)}^{⊺}\Phi_{R}\left(c_{i}\right)\right)}, \end{array}$$

where $\Phi_{R}\left(\cdot \right)$ refers to a representative vector for the surrounding context that includes the scene and neighboring characters, exp(·) denotes the exponential function, and $C_{\alpha ,l}$ indicates a set of characters that appeared in $s_{\alpha ,l}$. We use all the characters in our corpus to calculate the probability. To consider relationships between characters, Scene2Role2Vec [13] train role vectors by using the cross-entropy loss to approximate proximity degrees of the characters. However, for learning scene vectors, Scene2Role2Vec merely applies Graph2Vec, which only considers occurrences of social roles. On the other hand, Role2Vec and Scene2Vec do not directly consider the proximity degrees, since the proximity is already considered for composing the social roles. Also, learning relationships between characters enables the PV-DM part of Scene2Vec to consider whether a character interacted with other characters in a single conversation or multiple independent conversations. Bost et al. [39] and we [23] have already shown that distinguishing independent conversations affect the accuracy of interpreting scenes.

The PV-DBOW part of Scene2Vec predicts an occurrence probability of a character on a scene by using the inner product of their vector representations. An objective function of the PV-DBOW part is similar to the conventional PV-DBOW method. For a scene, we maximize an occurrence probability of every character that appeared on the scene and minimize the probability of characters that did not appear on the scene. This is formulated as:

(11) $$\begin{array}{cc} L_{\mathrm{SO}}\left(s_{\alpha ,l}\right)= & \sum_{\begin{array}{c} \forall c_{i}\in C_{\alpha ,l} \end{array}}\log P\left(c_{i}|\Phi \left(s_{\alpha ,l}\right)\right)≃\sum_{\begin{array}{c} \forall c_{i}\in C_{\alpha ,l} \end{array}}\frac{exp\left(\Phi {\left(c_{i}\right)}^{⊺}\Phi \left(s_{\alpha ,l}\right)\right)}{\sum_{\begin{array}{c} \forall c_{j} \end{array}}exp\left(\Phi {\left(c_{j}\right)}^{⊺}\Phi \left(s_{\alpha ,l}\right)\right)}. \end{array}$$

The PV-DBOW part of Scene2Vec is similar to Scene2Role2Vec. Only one difference is that Scene2Vec learns the occurrence of characters, and Scene2Role2Vec learns the occurrence of the social roles of the characters. However, as shown in our previous studies [12], characteristics of each narrative utterance are diluted in PV-DM approaches, since they focus on adjacency of utterances rather than each utterance. Thus, we have to emphasize the appearance of narrative utterances by using PV-DBOW parts. To integrate the PV-DM and PV-DBOW parts, we define an objective function of Scene2Vec as a summation of the objective functions of the two parts, as $L_{S}\left(s_{\alpha ,l}\right)=L_{\mathrm{SA}}\left(s_{\alpha ,l}\right)+L_{\mathrm{SO}}\left(s_{\alpha ,l}\right)$.

**Figure 5:**
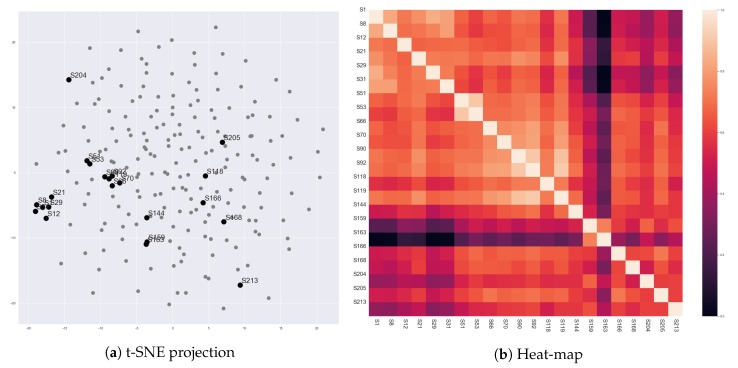
Vector representations of scenes in ‘The Godfather’ (1972) that were generated by Scene2Vec. Dots on (**a**) denote 213 scenes of ‘The Godfather’ (1972). Highlighted dots indicate scenes describing the meetings of the Corleone family. (**b**) presents a heat-map for similarity between scene vectors of the meeting scenes.

Figure 5 presents vector representations of 213 scenes in ‘The Godfather’ (1972), which were generated by Scene2Vec. We cannot examine all the scenes in our corpus due to their numerousness. Therefore, we selected scenes in ‘The Godfather’ (1972) that describe the meetings of the Corleone family (e.g., Scene # 1, # 8, # 12, # 21, # 29, # 31, # 51, # 53, # 66, # 70, # 90, # 92, # 118, # 119, # 144, # 159, # 163, # 166, # 168, # 204, # 205, and # 213). Since the scenes have the same subject, a comparison between their representations will expose whether scene vectors reflect the social relationships of characters in the scenes. Also, the comparison will show a correlation of the social relationships to the content of scenes.

First, Scene # 1, # 8, # 12, # 21, # 29, # 31, # 51, and # 53 commonly showed that ‘Don Vito Corleone’ ($c_{1}$) is the boss of the Corleone family. In these scenes, ‘Vito Corleone’ had high proximity for most of the other characters. However, Scene # 51 and # 53 contrasted the personality of ‘Sonny Corleone’ ($c_{3}$) with ‘Vito Corleone’. Dogmatic and emotional aspects of ‘Sonny’ were represented by high proximity for other characters on the character network. Also, on Scene # 21, ‘Michael Corleone’ attempted to keep distant from his family, and it was contrary to ‘Vito’ as a charismatic mafia boss. As shown in Figure 3, the distance was represented by the partitioned character network. The embedding results reflected these points. All the eight scenes were closely located, but Scene # 21, # 51, and # 53 were relatively distant from the other five scenes.

Scene # 66, # 70, # 90, # 92, and # 119 were between the assesination attempt for ‘Vito Corleone’ and the death of ‘Sonny Corleone’ ($c_{3}$). On these scenes, ‘Sonny’ lead the meetings, and ‘Michael’ started participating in the meetings. The five scenes were closely located, but Scene # 90, # 92, and # 119 were closer to each other than the other two scenes. In the three scenes, ‘Sonny’ and ‘Tom Hagen’ ($c_{4}$) had conflicts. For the crisis of the family, ‘Sonny’ reacted emotionally, but ‘Tom’ was careful. Thus, ‘Tom’ was more active (spoke more dialogues) in the three scenes than in the other scenes. This point could be reflected in the scene vectors.

On Scene # 159 and # 163, ‘Michael’ showed that he started to lead the Corleone family. Different from the earlier scenes, he took the lead of conversations. He showed high proximity to the other family members. Also, the two scenes were located closely and far from the other scenes. Scene # 204, # 205, and # 213 also exhibited ‘Michael Corleone’ as a cold-blooded mafia boss, since these scenes were describing that ‘Michael’ purged his enemies for the family safety. However, these three scenes were not close to Scene # 159 and # 163. This defect might come from that we considered only dialogues as interactions for constructing the character networks. However, in those scenes, the behaviors of characters describe most of the content. Additionally, Scene # 118, # 144, and # 166 were not closely located, although they had a common point that they showed ‘Vito Corleone’ after he was weakened. The weakening of ‘Vito Corleone’ might also be described by his facial and vocal expressions, rather than the number of his dialogues.

### 3.3. Representing Stories Based on the Order of Scenes

Finally, Story2Vec-H concentrates on that a story is a series of events (scenes). The content of a story is exposed by which events are included in the story. However, how the events are arranged and organized reflects storytelling strategies for delivering the events. Figure 3a–c provides an example of the content of the story in ‘The Godfather’ (1972) that was made by the order of scenes. We already described the content of the three scenes (Scene # 21, # 92, and # 163). These scenes commonly showed the family meeting of the Corleone family. On Scene # 21, ‘Michael Corleone’ ($c_{2}$) was not involved in the family business. Then, he started participating in the meeting as a reaction to the tragedy in his family. Finally, Scene # 163 described ‘Michael’ as a mafia boss. Due to the order of scenes, users can understand that ‘Michael’ changed from a civilian to a mafia boss to keep his family. Also, the order made inner conflicts of ‘Michael’ probable, which were between his belief and the crisis of his family. If this movie attempted to tell a story about a mafia boss that wants to get out of the family business, these scenes would be in the reverse order.

To learn the arrangement of scenes, the adjacency between scenes is determined by their sequential order. With a fixed-length window $W_{L}$, neighborhoods of a scene $s_{\alpha ,l}$ consist of scenes from $s_{\alpha ,l-W_{L}}$ to $s_{\alpha ,l+W_{L}}$. This neighborhood is called ‘sequential neighborhood,’ and can be formulated as:

(12) $$\begin{array}{cc} N^{S}\left(s_{\alpha ,l}\right)= & \{s_{\alpha ,l+\Delta L}\;|\;\left|\Delta L\right|\leq W_{L},s_{\alpha ,l+\Delta L}\neq s_{\alpha ,l}\}. \end{array}$$

It is also illustrated by the upper part of Figure 2.

Since scenes in stories are sequentially ordered as words in documents, the PV-DM part of Story2Vec-H simply applies the conventional PV-DM method. As with Scene2Vec, Story2Vec-H does not use the negative sampling. There are two main reasons. First, scenes are intrinsic and not shared among narrative works. Also, the number of scenes in narrative works is not as enormous as the number of words in documents; on average, a movie contains 140 to 160 scenes.

Therefore, in the PV-DM part, a co-occurrence probability of a scene is estimated based on its neighboring scenes and a story that contains the scene. The surrounding context of the scene is integrated by using an average of their vector representations. Thereby, its objective function is designed to maximize a co-occurrence probability of scenes that are closely located to a target scene and minimize the probability for scenes that are not adjacent to the scene. This is formulated as:

(13) $$\begin{array}{cc} L_{\mathrm{HA}}\left(C_{\alpha }\right)= & \sum_{\begin{array}{c} 1\leq l\leq L \end{array}}\log P\left(s_{\alpha ,l}|N^{S}\left(s_{\alpha ,l}\right),\Phi \left(C_{\alpha }\right)\right)≃\sum_{\begin{array}{c} 1\leq l\leq L \end{array}}\frac{exp\left(\Phi {\left(s_{\alpha ,l}\right)}^{⊺}\Phi_{H}\left(s_{\alpha ,l}\right)\right)}{\sum_{\begin{array}{c} \forall s_{\alpha ,m} \end{array}}exp\left(\Phi {\left(s_{\alpha ,m}\right)}^{⊺}\Phi_{H}\left(s_{\alpha ,l}\right)\right)}, \end{array}$$

where $\Phi_{H}\left(\cdot \right)$ denotes a representative vector for surrounding context including neighboring scenes and a story. Story2Vec-F and the PV-DM part of Story2Vec-H commonly learn time-sequential features of stories. In Story2Vec-F, the features are reflected by dynamic changes in structures of character networks. On the other hand, the PV-DM part of Story2Vec-H is based on the content of scenes and their arrangement, although representations of the scenes also come from the character networks.

As with the PV-DM part, the PV-DBOW part merely uses the conventional PV-DBOW method. An occurrence probability of a scene in a story is predicted based on the softmax function and an inner product of their vector representations. We maximize an occurrence probability of every scene that is included in the story and minimize the probability for scenes that are not in the story. This is formulated as:

(14) $$\begin{array}{cc} L_{\mathrm{HO}}\left(C_{\alpha }\right)= & \sum_{\begin{array}{c} 1\leq l\leq L \end{array}}\log P\left(s_{\alpha ,l}|\Phi \left(C_{\alpha }\right)\right)≃\sum_{\begin{array}{c} 1\leq l\leq L \end{array}}\frac{exp\left(\Phi {\left(s_{\alpha ,l}\right)}^{⊺}\Phi \left(C_{\alpha }\right)\right)}{\sum_{\begin{array}{c} \forall s_{\alpha ,m} \end{array}}exp\left(\Phi {\left(s_{\alpha ,m}\right)}^{⊺}\Phi \left(C_{\alpha }\right)\right)}. \end{array}$$

Story2Vec-D and the PV-DBOW part of Story2Vec-H commonly learn static features of a story. The PV-DBOW part of Story2Vec-H aims to represent which scenes compose the story. We expect that our scene vectors and story vectors reflect the content of scenes and stories, respectively. On the other hand, Story2Vec-D is directly based on the appearance of substructures of character networks (social roles). To integrate the PV-DM and PV-DBOW parts, we define an objective function of Story2Vec-H as a summation of the objective functions of the two parts, as $L_{H}\left(C_{\alpha }\right)=L_{\mathrm{HA}}\left(C_{\alpha }\right)+L_{\mathrm{HO}}\left(C_{\alpha })\right)$.

**Figure 6:**
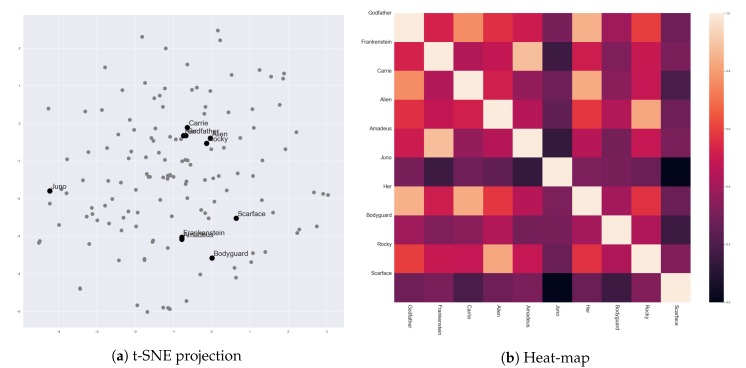
Vector representations of stories in movies that were generated by Story2Vec-H. Dots on (**a**) indicate 142 movies in our corpus. Highlighted dots denote movies selected for discussion. (**b**) presents a heat-map for similarity between story vectors of the ten movies.

Figure 6 presents vector representations of stories in 142 movies, which were generated by Story2Vec-H. We selected ten movies from our movie corpus to discuss whether the results of Story2Vec-H are in accord with our expectations. Table 2 presents titles and metadata of the selected movies. Simply speaking, the story vectors of Story2Vec-H seems highly affected by changes in the social relationships of protagonists.

As discussed in the previous sections, ‘The Godfather’ (1972) contains the two round characters. Scenes in ‘The Godfather’ presented gradual changes in ‘Vito’ and ‘Michael Corleone’ in opposite directions. Similar to ‘Michael’, ‘Her’ (2013) described changes of ‘Theodore’ from a passive to an active character. As ‘Michael’ became intimate with his family members, ‘Theodore’ were getting higher proximity with ‘Samantha’. ‘Carrie’ in ‘Carrie’ (1976) showed changes in reactions for her mother and boyfriend. These changes accompanied the growth of social relationships with friends in her school. These three movies got similar vector representations, as shown in Figure 6. ‘Rocky’ in ‘Rocky’ (1976) also showed a change in the same direction. However, his social relationships mainly changed in emotional areas, not clearly reflected by the interaction frequency. Also, in ‘Alien’ (1979), ‘Ripley’ changed from a passive to an active character. Characters that had relationships with ‘Ripley’ were getting less since the characters kept dead. Thus, the locations of ‘Rocky’ (1976) and ‘Alien’ (1979) were nearby the above three movies, but not as close as among the three of them.

Similarly, ‘Amadeus’ (1984) and ‘Frankenstein’ (1994) presented the one-way change, but the direction of the change was opposite to the above movies. ‘Wolfgang Amadeus Mozart’ in ‘Amadeus’ (1984) kept losing his social relationships, as with his reputation and power. ‘Frankenstein’ (1994) also described that ‘Dr. Victor Frankenstein’ was losing his social relationships by the death and leaving of acquaintances. The loss accompanied changes in his personalities from a haughty to a wimpish character. Therefore, these two movies were closely located and far from ‘The Godfather’ (1972), ‘Her’ (2013), and ‘Carrie’ (1976).

A few movies showed multiple changes in their leading characters. ‘Scarface’ (1983) described a process that ‘Tony Montana’ became the boss of a crime organization. In the beginning, ‘Tonny’ was introvert, but he became hot-blooded and violent after being the boss. However, ‘Scarface’ (1983) presented one more change. At the ending, ‘Tonny’ lost his achievement, and his interactions became infrequent again. ‘Juno’ (2013) also showed two-stage changes. Social relationships of ‘Juno MacGuff’ with her family became better than the beginning. However, her relationship with her boyfriend was getting worse until the climax, and then getting better again. ‘The Bodyguard’ (1992) presented more changes than ‘Scarface’ (1983) and ‘Juno’ (2013). Among its two leading characters (‘Frank Farmer’ and ‘Rachel Marron’), only ‘Rachel Marron’ exhibited a change from a stubborn to a kind character. Nevertheless, their relationship started from a conflict, fell in love, got another conflict, and became positive again. Therefore, these three movies were distant from the other movies, but also not close to each other.

For designing the Story2Vec-H method, we expected that this method reflects events in scenes and the order of the scenes more distinctly. As shown in the above examples, story vectors generated by Story2Vec-H were close to representations of changes in round characters. However, the changes in characters are caused by conflicts described in the scenes. Thus, we can say that Story2Vec-H reflects the characteristics and arrangements of the conflicts, at least indirectly.

Finally, we can integrate the objective functions of three parts of the hierarchical story embedding model as:

(15) $$\begin{array}{c} L_{J}\left(C_{\alpha }\right)=L_{H}\left(C_{\alpha }\right)+\sum_{\begin{array}{c} 1\leq l\leq L \end{array}}\left[L_{S}\left(s_{\alpha ,l}\right)+\sum_{\begin{array}{c} \forall c_{i}\in C_{\alpha }\\ N\left(s_{\alpha ,l}\right)∋a_{i,i}\neq 0 \end{array}}L_{C}\left(c_{i}\right)\right]. \end{array}$$

In the same manner, the layers for hierarchical representations can bemore subdivided. Also, these layers are able to be replaced according to media, formats, or genres of narrative works. However, adding more layers has an abstruse issue that narrative utterances excluding social roles (i.e., substructures of character networks) are not shared among narrative works. Simply speaking, it is difficult to provide enough learning opportunities for each utterance. Although we present a case with only the four layers (e.g., social roles, characters, scenes, and stories), this issue will hinder the quality of vector embedding as the proposed model getting deeper.

## 4. Evaluation

Section 1 has presented several research questions about the fundamental assumptions of this study. We attempted to verify the research questions and the effectiveness of the proposed methods: Char2Vec, Scene2Vec, and Story2Vec-H. For the validation, we mainly compared the proposed methods with the existing ones (i.e., Role2Vec [12], Scene2Role2Vec [13], and Story2Vec-F/D/U [12]) in terms of their accuracy for measuring similarity between narrative utterances.

**Figure 7:**
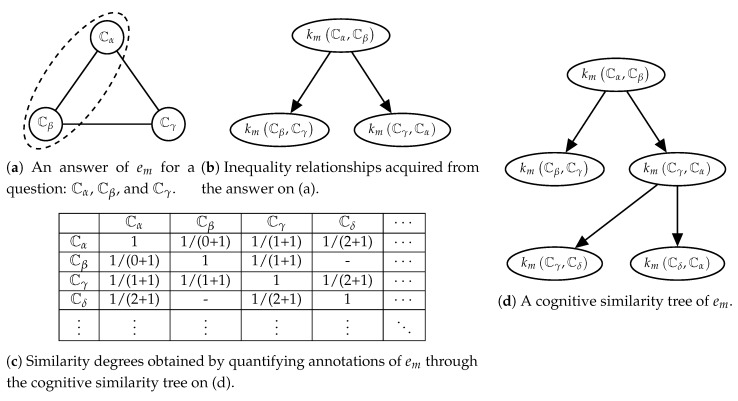
Procedures for collecting the cognitive similarity. Circular and elliptical nodes indicate movies and similarity degrees between the movies, respectively. (**a**) presents that each evaluator chose two of three movies that are more similar to each other than the remaining one. From the answer on (**a**), we can extract two inequality relationships between similarity degrees, as shown in (**b**). (**d**) is an example of the cognitive similarity tree that is composed by aggregating the inequality relationships acquired from each evaluator. We quantify the similarity degrees using their depth on the tree, as displayed in (**c**). The data collection procedures for the other narrative utterances are the same as the procedures for movies.

It is difficult to acquire ground truth data for the similarity of narrative utterances. For general users, quantifying the similarity is a challenging task. For example, the climax scenes of ‘The Godfather’ (1972) and ‘Joker’ (2019) are similar to as much as 0.40. Also, we cannot assure that the users will measure the similarity with consistent criteria. Therefore, our previous study [12] has proposed a method for lightening users’ burden in collecting the similarity degrees. This method asks a user to pick two of three narrative utterances, which are more similar than another one. Thus, each answer includes inequality relations between the similarity degrees. Let suppose that we asked a question about $C_{\alpha }$, $C_{\beta }$, and $C_{\gamma }$. When a user selected $C_{\alpha }$ and $C_{\beta }$, this answer indicates that $k\left(C_{\alpha },C_{\beta }\right)$ is bigger than $k\left(C_{\beta },C_{\gamma }\right)$ and $k\left(C_{\gamma },C_{\alpha }\right)$. By aggregating these answers from each user, we can build a tree (called ‘cognitive similarity tree’ [12]), which has the similarity degrees as nodes and the inequality relationships as directed edges. Finally, we quantify the similarity degrees based on their depth on the tree. When depth of $k\left(C_{\beta },C_{\gamma }\right)$ on a tree of the user $u_{m}$ is D, $k_{m}\left(C_{\beta },C_{\gamma }\right)$ (i.e., $k\left(C_{\beta },C_{\gamma }\right)$ measured by $u_{m}$) is equal to $\frac{1}{D+1}$. We call the similarity degrees measured by the tree as ‘cognitive similarity’. Detailed procedures of this data collection method are illustrated in Figure 7 and described in our previous study [12].

Based on this method, we collected human perception for the similarity between protagonists, scenes, and stories in real movies. The experimental subjects and procedures for preprocessing them are the same as the previous study [12]. We acquired scripts and metadata of 142 movies from IMSDb (https://www.imsdb.com/) and IMDB (https://www.imdb.com/). Character networks of the movies were extracted from their scripts by using CharNet-Extractor [24]. A list of the experimental subjects and our implementations for the story embedding models are available on GitHub (https://github.com/O-JounLee/Story2Vec). Furthermore, the proposed methods were implemented in Python by modifying open source implementations of Graph2Vec (https://github.com/MLDroid/graph2vec_tf), Subgraph2Vec (https://github.com/MLDroid/subgraph2vec_tf), and Story2Vec-F/D/U.

For the data collection, we composed a human evaluator group that consisted of 65 people who have been faculty members and students of Chung-Ang University, Pohang University of Science and Technology, and Inha University. From the 65 human evaluators, we collected 1066 answers for the character similarity, 743 answers for the scene similarity, and 1471 answers for the story similarity. These answers were transformed into the similarity degrees by using the cognitive similarity tree for each evaluator. When multiple users answered for a similarity degree, these answers were aggregated by the arithmetic mean; $k\left(C_{\alpha },C_{\beta }\right)=\frac{1}{\left|K_{\alpha ,\beta }\right|}\sum k_{m}\left(C_{\alpha },C_{\beta }\right)$. In here, $K_{\alpha ,\beta }$ is the set of annotations for similarity between $C_{\alpha }$ and $C_{\beta }$. Then, we used mean absolute errors (MAEs) to compare this ground-truth dataset with similarity degrees measured by the embedding models. Where $E\left(C_{\alpha },C_{\beta }\right)=\left|k\left(C_{\alpha },C_{\beta }\right)-\hat{k}\left(C_{\alpha },C_{\beta }\right)\right|$ is the absolute error for the similarity degree between $C_{\alpha }$ and $C_{\beta }$, and K is the set of all the collected similarity degrees, MAE is calculated by $\frac{1}{\left|K\right|}\sum E\left(C_{\alpha },C_{\beta }\right)$.

Furthermore, the proposed model requires various hyper-parameters. This study concentrates on validating whether the inherent hierarchical structures of stories are useful for representing various narrative utterances. Therefore, our contribution is mainly on (i) computational definitions of the hierarchical structures and (ii) representation learning methods based on the definitions. Excluding these points, the proposed model shares the same approach with our previous study [12], which applies the WL relabeling and Word2Vec-based models on the character network, as well as the experimental subjects. Therefore, we reused the hyper-parameters, which are empirically tuned in our previous study. Most of the parameters of the conventional Graph2Vec model were set as: the number of epochs η=80, the learning rate ρ=0.025, the number of dimensions δ=32, the maximum degree of social roles D=4, and the number of negative samples k=37.

We conducted a grid search for the hyper-parameters required by the proposed model: the weighting factor for the noise distribution of social roles *w* (0.00 to 1.00 with a step of +0.10), the window size for degrees of social roles $W_{D}$ in Equation (6) (1 to *D* = 4 with a step of +1), the window size for scenes $W_{T}$ in Equation (6) (1 to 5 with a step of +1), and the window size for scenes $W_{L}$ in Equation (12) (1 to 5 with a step of +1). We evaluated these cases based on the average accuracy of the proposed model (i.e., MAE for all the Char2Vec, Scene2Vec, and Story2Vec-H). The HSE model exhibited the highest performance on: w=0.90, $W_{D}=1$, $W_{T}=2$, and $W_{L}=4$.

### 4.1. Accuracy of Role2Vec and Char2Vec for Identifying Roles of Characters

We validated RQ 2 and exhibited the effectiveness of Char2Vec by comparing its accuracy for measuring similarity between protagonists in movies with the accuracy of Role2Vec. The evaluators are difficult to remember all the characters, including extras. We collected cognitive similarity degrees only between the protagonists of the movies. Then, we estimated the character similarity by using a combination of cosine similarity and Euclidean distance between the character vectors. This can be formulated as:

(16) $$\begin{array}{c} \hat{k}\left(c_{i},c_{j}\right)=\frac{\Phi^{*}{\left(c_{i}\right)}^{⊺}\Phi^{*}\left(c_{j}\right)}{∥\Phi^{*}\left(c_{i}\right)∥∥\Phi^{*}\left(c_{j}\right)∥}\times \frac{1}{∥\Phi^{*}\left(c_{i}\right)-\Phi^{*}\left(c_{j}\right)∥+1}, \end{array}$$

where $∥\cdot ∥$ indicates the Euclidean norm and $\hat{k}\left(c_{i},c_{j}\right)$ refers to a computationally estimated similarity between two characters $c_{i}$ and $c_{j}$. Equation (16) was also used for scenes and stories. Subsequently, the character similarity was evaluated by comparing it with the cognitive similarity.

**Figure 8:**
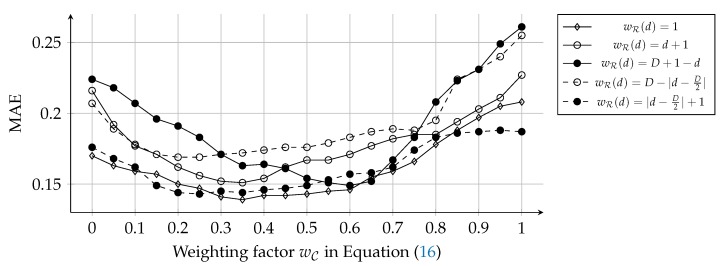
Accuracy of the character similarity according to the weighting factors, $w_{C}$ and $w_{R}\left(d\right)$.

**Figure 9:**
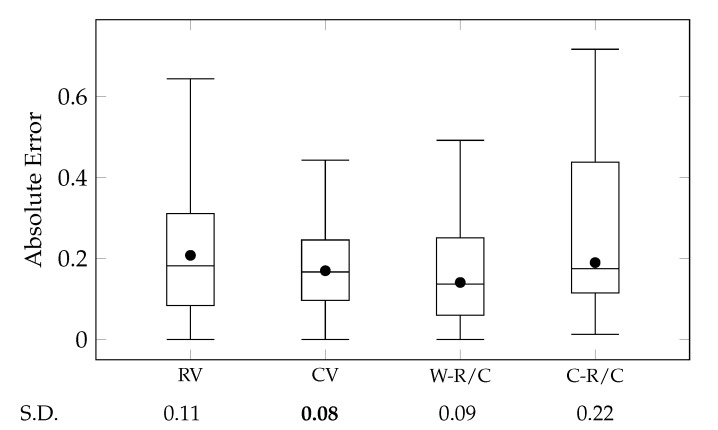
Accuracy for measuring the similarity of protagonists in the movies. W-R/C and C-R/C refer to the weighted average and concatenation cases, respectively. Three horizontal lines indicate first quartiles, median values, and third quartiles of absolute errors, respectively. Tops and bottoms of whiskers refer to maxima and minima of absolute errors, respectively. Circular dots denote mean absolute errors (MAEs) (as with averages of absolute errors). Lastly, S.D. under the labels of plots indicates the standard deviations of absolute errors in each case.

Since Char2Vec and Role2Vec reflect dynamic and static features, respectively, they can complement the weakness of each other. Various methods can be used for combining the results of Char2Vec and Role2Vec. First, we can adjust the significance levels of the dynamic and static features using the weighted average (W-R/C). This can be formulated as:

(17) $$\begin{array}{c} \Phi^{*}\left(c_{i}\right)=\left(1-w_{C}\right)\times \Phi \left(c_{i}\right)+w_{C}\times \frac{1}{\sum_{0\leq d\leq D}w_{R}\left(d\right)}\times \sum_{0\leq d\leq D}w_{R}\left(d\right)\times \Phi \left(c_{i,L}^{\left(d\right)}\right), \end{array}$$

where $w_{C}$ is a weighting factor for how much we consider the static features, and $w_{R}\left(d\right)$ is a weighting factor for role vectors according to the degree. For $w_{R}\left(d\right)$, we compared five cases: $w_{R}\left(d\right)=1$, $w_{R}\left(d\right)=d+1$, $w_{R}\left(d\right)=D+1-d$, $w_{R}\left(d\right)=D-\left|d-\frac{D}{2}\right|$, and $w_{R}\left(d\right)=\left|d-\frac{D}{2}\right|+1$. The first case indicates that all the degrees are equally significant. The other four cases emphasize high degrees, low degrees, middle degrees, and extreme (both high and low) degrees, respectively. We conducted a hyper-parameter search to find the optimal $w_{C}$ and $w_{R}\left(d\right)$, as displayed in Figure 8. Second, the concatenation is widely-used for merging vectors as much as the average. This case is notated as ‘C-R/C.’ We compared these two cases with cases that the character vector (CV) and role vector (RV) were solely used. Since characters have social roles on every scene and degree, the RV case only uses role vectors of social roles on the denouement, as with the previous study [12]. Figure 9 illustrates comparisons between these cases using a box-and-whisker plot for their absolute errors.

**Figure 10:**
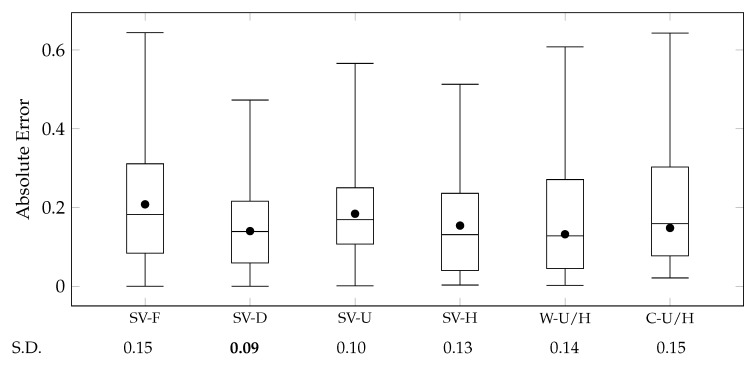
Accuracy for measuring the similarity of stories in the movies. W-U/H and C-U/H refer to the weighted average and concatenation cases, respectively.

A comparison between RV and CV cases exhibited that the proposed method (Char2Vec) can generate more accurate representations of characters than directly using representations of their social roles (Role2Vec). The role vectors are trained by the temporal and radial adjacency of social roles. And, Char2Vec aggregates roles vectors of a character based on temporal adjacency and appearances of the social roles. Therefore, this study claims the contribution to that Char2Vec provides a reasonable aggregation method rather than a performance improvement to Role2Vec. Nevertheless, interestingly, Char2Vec improved the quality of vector representations in terms of both accuracy and variance. This result underpins RQ 2 that dynamic changes in the social roles of characters are more effective in representing typicality of character roles than static compositions of social roles. Additionally, the comparison between RV and CV cases is opposite to experimental results for story vectors, which were presented in our previous study [12] (in Figure 10). In the former experiment, static social roles on the denouement (Story2Vec-D) outperformed dynamic changes in the social roles according to narrative time (Story2Vec-F).

The W-R/C and C-R/C cases embrace both dynamic and static features of character roles. The experimental results in Figure 8 and Figure 9 show that the two kinds of features compensate the defect of each other through their two combinations (W-R/C and C-R/C). The W-R/C case exhibited lower MAEs than both the CV and RV cases. Also, the C-R/C case demonstrated a lower MAE than the RV case. However, the variance in the two cases was worse than the original role vectors and character vectors. Concerning that Char2Vec exhibited a lower variance than Role2Vec, the combination of various vector representations requires a more sophisticated approach than mere average or concatenation.

As shown in Figure 8, the hyper-parameter search was conducted for a range $w_{C}\in \left[0,1\right]$ with a step size +0.05. The weighting function was tested for five cases that weight uniformly (1), on high (d+1), low (D+1−d), medium ($D-|d-\frac{D}{2}|$), and extreme degrees ($|d-\frac{D}{2}|+1$), respectively. The uniform case ($w_{R}\left(d\right)=1$) had the lowest MAE at the most of $w_{C}$ values, among the weighting functions. And, when $w_{C}=0.35$ and $w_{R}\left(d\right)=1$, the W-R/C case had the lowest MAE. This result indicates that character vectors are more effective for representing characters than role vectors, but role vectors also supplement the accuracy of the character representation. Also, every degree of social roles is equally (at least, similarly) important. The extreme case ($w_{R}\left(d\right)=\left|d-\frac{D}{2}\right|+1$) exhibited the similar performance with the uniform case. Among the other three cases, the low and medium cases ($w_{R}\left(d\right)=D+1-d$ and $=D-|d-\frac{D}{2}|$) showed the best and worst performance, respectively. These results could come from that centrality of characters affects human perception for characters more than we expected, since social roles on D=0 are equal to centrality-based categories (i.e., protagonist, main, minor, and extra characters). Also, high degrees might represent character roles more detailedly than medium degrees. We can apply other types of kernel functions (e.g., Epanechnikov, Quartic, and Gaussian kernels) for adjusting the weighting factors. However, complicated kernels are not realistic, since character networks are far smaller than social networks in the real world and do not require the large maximum degree (*D*).

Furthermore, the concatenation (C-R/C) could not outperform the weighted average (W-R/C). This result was also unexpected since the concatenation has usually exhibited a better performance in the existing studies for the vector embedding [18,19]. The reason could be that Equation (16) has not considered the importance of each component within vector representations, while the existing studies mostly evaluated their embedding based on linear classifiers. Therefore, the performance evaluation of other various tasks will be required for further research. With the experimental results, we verified RQ 2 and the effectiveness of Char2Vec. At the same time, we showed that using Char2Vec and Role2Vec together improves their accuracy.

### 4.2. Accuracy of Scene2Vec and Scene2Role2Vec for Measuring Similarity between Scenes

This section validates RQ 3 by comparing Scene2Vec based on characters in each scene with Scene2Role2Vec [13] based on social roles in each scene. The scene embedding models were evaluated by their accuracy for estimating similarity between scenes. The accuracy was measured based on similarity annotated by the evaluators. However, for regular users, it is difficult to remember all the scenes in movies. Therefore, we restricted scenes into major scenes that would be remembered by the evaluators.

Nevertheless, we do not have a concrete method for finding the major scenes, while we can discover the protagonists by using their node centrality. Before collecting the scene similarity, we asked the evaluators to annotate major scenes in movies that they watched. For the 142 movies in our corpus, the evaluators annotated 274 scenes (1.93 scenes for each movie on average). Each annotation consisted of descriptions for a scene in the natural language (e.g., for the movie ‘The Godfather’ (1972), “A scene describing a business meeting of ‘Don Vito Corleone’ during a wedding ceremony of his daughter.”). Since descriptions of the evaluators are different even for the same scene, we manually found the 274 major scenes from the movies according to 611 annotations. Also, during the similarity collection, we had to enable the evaluators to identify the scenes. We provided the descriptions to the evaluators with keyframes of the major scenes.

**Figure 11:**
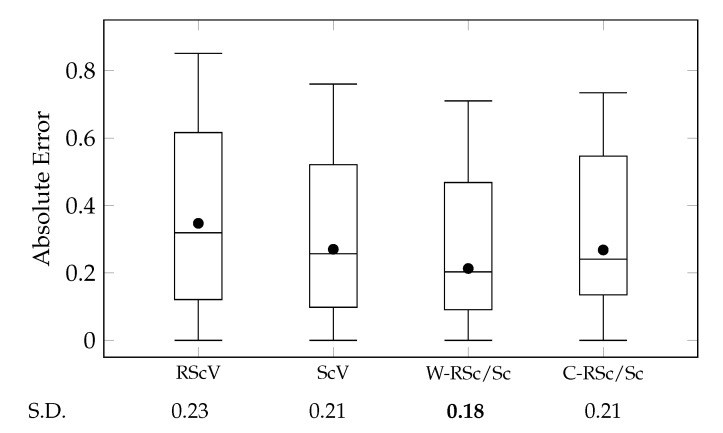
Accuracy for measuring the similarity between scenes in the movies. W-RSc/Sc and C-RSc/Sc refer to the weighted average and concatenation cases, respectively.

Moreover, scene vectors of Scene2Role2Vec are close to representations of structures of character networks on each scene. We examined whether these structural features are helpful for representing the content of scenes by aggregating the two kinds of scene vectors. As with the former experiment, we aggregated the scene vectors using the weighted average (W-RSc/Sc) and the concatenation (C-RSc/Sc). The W-RSc/Sc case combines scene vectors generated by the two methods with a weighting factor $w_{\mathrm{SC}}\in \left[0,1\right]$. This can be formulated as:

(18) $$\begin{array}{c} \Phi^{*}\left(s_{\alpha ,l}\right)=w_{\mathrm{SC}}\times \Phi^{R}\left(s_{\alpha ,l}\right)+\left(1-w_{\mathrm{SC}}\right)\times \Phi^{H}\left(s_{\alpha ,l}\right), \end{array}$$

where $\Phi^{R}\left(s_{\alpha ,l}\right)$ and $\Phi^{H}\left(s_{\alpha ,l}\right)$ indicate scene vectors of $s_{\alpha ,l}$ generated by Scene2Role2Vec and Scene2Vec, respectively. We compared the aggregations with the cases that Scene2Vec and Scene2Role2Vec were solely used (ScV and RScV). Figure 11 presents comparisons between these cases by using a box-and-whisker plot for their absolute errors.

The experimental results exhibited that Scene2Vec outperformed Scene2Role2Vec [13]. The ScV case exhibited lower MAE and more stable absolute errors than the RScV case. These results validate that appearances and relationships of characters are more effective in representing the content of scenes than structures of character networks on each scene (RQ 3). Also, although Scene2Vec does not directly consider time-sequential features of stories, these features are already reflected by character vectors on Char2Vec and propagated from Story2Vec-H during its learning procedures. However, the gap of standard deviation between the ScV and RScV was not significant. This point is different from the other experiments that compared time-aware methods with non-time-aware ones (e.g., Story2Vec-F/D [12] and Char2Vec/Role2Vec). Although most of the narrative utterances have dynamicity, how much the dynamicity affects human perception for the narrative utterances could be different.

Furthermore, the range of similarity degrees is [0,1]. MAE nearby 0.50 indicates that a similarity measurement cannot distinguish whether two narrative utterances are similar. Therefore, it is difficult to say that Scene2Vec and Scene2Role2Vec performed satisfactory accuracy. Methods for embedding other narrative utterances (Role2Vec, Char2Vec, and Story2Vec-F/D/U/H) exhibited much lower MAE than the scene embedding methods, as displayed in Figure 9 and Figure 10. The two methods commonly focused on relationships and appearances of characters on each scene. These results say that we should find more other features for representing events described by scenes.

**Figure 12:**
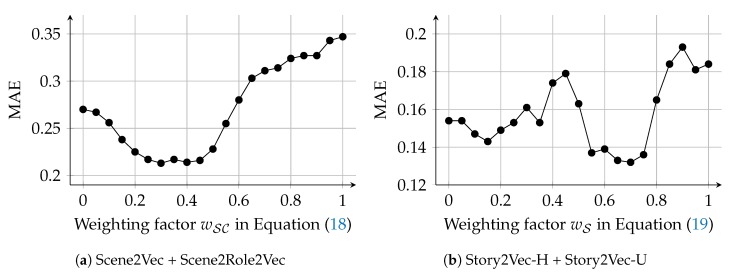
Accuracy of the scene similarity and story similarity according to the weighting factors.

We examined whether aggregating scene vectors generated by Scene2Vec and Scene2Role2Vec outperforms the cases that the methods were used alone (ScV and RScV). The W-RSc/Sc case exhibited the lowest MAE and variance of absolute errors among all the cases. Nevertheless, in terms of the variance, the improvement was not vivid. The W-RSc/Sc case employs the weighting factor $w_{\mathrm{SC}}$ in Equation (18) to adjust the influence of the two embedding methods. We searched the optimal $w_{\mathrm{SC}}$ in a range [0,1] with a step size +0.05, as displayed in Figure 12a. The W-RSc/Sc case exhibited the lowest MAE on $w_{\mathrm{SC}}=0.30$. However, the accuracy did not change significantly according to the changes in $w_{\mathrm{SC}}$. Therefore, it is difficult to say which scene embedding method has more contributions to the W-RSc/Sc case. MAE of the C-RSc/Sc case was lower than of the RScV case but slightly higher than of the ScV case. In terms of the variance, the C-RSc/Sc case exhibited a little lower variance than the ScV case. Combining the two methods improved the quality of story vectors. However, the improvement was insignificant.

These results could come from that Scene2Vec and Scene2Role2Vec are not much different from each other. To examine this notion, we measured the Pearson correlation coefficient (PCC) and the mean absolute deviation (MAD) between the scene similarity measured by the two methods. When PCC is equal to 1.00, the results of the two methods have the same tendency. And, MAD is 0.00, if the two kinds of scene similarity degrees are the same. PCC and MAD were 0.84 and 0.12, respectively. We assumed that the composition of characters is more appropriate to represent events on scenes than the composition of the temporal roles of the characters. However, there was no significant difference.

### 4.3. Accuracy of Story2Vec Models for Measuring Story Similarity

We verified RQ 4 and evaluated the effectiveness of Story2Vec-H, based on the accuracy of story vectors generated by Story2Vec-H. We exhibited the features of Story2Vec-H (i.e., order and composition of scenes) are effective for representing stories by comparing the accuracy of its story vectors with vectors generated by the existing methods: Story2Vec-F, Story2Vec-D, and Story2Vec-U [12]. The accuracy of story vectors was assessed by measuring the similarity between stories and comparing the estimated similarity with human perception.

The HSE model reflects the hierarchical structures of stories, and Story2Vec-H especially aims to represent which scenes are in the stories with which order. On the other hand, the existing methods [12] represent stories by using structural features of character networks. Thus, a combination of these two kinds of methods will be able to improve the accuracy of story representations. First, we suggest the weighted average of story vectors generated by Story2Vec-U and Story2Vec-H (W-U/H). This can be formulated as:

(19) $$\begin{array}{c} \Phi^{*}\left(C_{\alpha }\right)=w_{S}\times \Phi^{U}\left(C_{\alpha }\right)+\left(1-w_{S}\right)\times \Phi^{H}\left(C_{\alpha }\right), \end{array}$$

where $\Phi^{U}\left(C_{\alpha }\right)$ and $\Phi^{H}\left(C_{\alpha }\right)$ denote story vectors of $C_{\alpha }$ generated by Story2Vec-U and Story2Vec-H, respectively. By adjusting the weighting factor $w_{S}\in \left[0,1\right]$, we can also examine the contributions of the two methods to estimating the story similarity. We searched the optimal $w_{S}$ in a range of [0,1] with a step of +0.05, as displayed in Figure 12b. The concatenation is also a widely-used approach for combining feature vectors. C-U/H indicates a case that we concatenated story vectors generated by the Story2Vec-U and Story2Vec-H. We compared the combinations with the cases that the four embedding models (Story2Vec-F/D/U/H) were solely used. Figure 10 presents the results of this experiment by the box-and-whisker plot.

The experimental results demonstrated that Story2Vec-H outperformed Story2Vec-F in terms of the accuracy of the average and variance. Also, although Story2Vec-H exhibited a lower MAE than Story2Vec-U, Story2Vec-H had a higher variance of the absolute errors. However, Story2Vec-D showed lower MAE and variance of the errors than Story2Vec-H. These results are difficult to be explicit validation of RQ 4. Story2Vec-F and Story2Vec-D are based on dynamic and static features of stories, respectively. And, Story2Vec-H and Story2Vec-U represent both the dynamic and static features. Also, the PV-DBOW part of Story2Vec-H considers all the scenes individually, different from that Story2Vec-D reflects overviews of the entire story. We reconfirmed the notion from our previous study [12] that human perception for the story is close to the overview rather than a series of events.

RQ 4 deals with features that the embedding methods foot on—(i) order and composition of scenes (Story2Vec-H) and (ii) structures of character networks (Story2Vec-F/D/U). The experimental results cannot underpin that a feature significantly outperforms the other one. They signify that dealing with scenes individually is not helpful for representing stories. However, the proposed model is designed to handle narrative utterances individually. Although this study did not conduct experiments on diverse narrative utterances, the problem can also be on other utterances between the scene and story layers (e.g., sequences and acts).

For RQ 1, we attempted to validate that hierarchical structures of narrative works are effective for learning representations of narrative utterances on various granularity levels. Char2Vec outperformed the existing method on the character layer. However, on the scene layer, Scene2Vec and Scene2Role2Vec [13] could not exhibit the high performance commonly, as shown in Figure 11. Also, Story2Vec-H exhibited similar performance to the existing methods. The hierarchies of narrative utterances are still valuable in terms of that they enable us to embed narrative utterances, which have not got interests from the existing methods. At the same time, we have to look for ways to improving the performance of the proposed HSE model.

Furthermore, hierarchical structures of stories are a part of common sense, as well as their time-sequential features [20,21,40,41,42]. The computational narrative analysis is not only for user applications [7,8] but also for analysis itself for supporting literature or narratology studies [43,44]. For this purpose, we have to look inside stories, not treating the stories monolithic.

Combinations of Story2Vec-H and Story2Vec-U outperformed cases that the methods were solely used, in terms of the MAE. Nevertheless, the variance of absolute errors was higher in the combinations than in the standalone cases. First, the W-U/H case employs the weighting factor (in Equation (19)) to combine story vectors from the two methods. The optimal $w_{S}$ has been discovered by searching a range of [0,1] with a step of +0.05, as displayed in Figure 12b. The W-U/H case exhibited the lowest MAE at $w_{S}=0.70$. Also, from $w_{S}=0.55$ to 0.75, the W-U/H case showed relatively consistent MAE. When $w_{S}$ is close to 0.00, the W-U/H case is as with the SV-H case. Thus, Story2Vec-U had more contributions to the W-U/H case than Story2Vec-H, although Story2Vec-H has a lower MAE than Story2Vec-U. Although the C-U/H case also performed a lower MAE than the SV-H and SV-U cases, the W-U/H case outperformed this case in terms of both the average and variance of absolute errors.

The story embedding methods have been designed to represent their own narrative characteristics. Each component of story vectors represents an unknown narrative feature. Even the vector components generated by different embedding methods will have different meanings. Thus, in the W-U/H case, simply averaging them might cause a high variance. However, the C-U/H case is not explained by this reason. As discussed for Role2Vec and Char2Vec, our evaluation method does not consider the significance of each vector component. Simply concatenating vector representations will accrue losses of vector components, as well as gains from them. In further studies, we have to find other tasks for the unbiased evaluation.

In conclusion, Story2Vec-H exhibited that the order and composition of scenes in a story are effective for representing stories, as well as structures of character networks (RQ 4). A comparison between Story2Vec-H and Story2Vec-U also showed the effectiveness of hierarchies of narrative utterances for representing themselves (RQ 1). Lastly, Story2Vec-F suffered from overly numerous social roles for each narrative work. Comparing Story2Vec-F with Story2Vec-H, this problem looks dispersed into multiple layers in the proposed model.

## 5. Conclusions

This study concentrated on learning hierarchical representations of narrative utterances on various granularity levels. We proposed an embedding model that learns the inclusion and adjacency between the narrative utterances in their hierarchies. This model can be extended to various utterances from a shot to a transmedia franchise. However, we only use four layers (e.g., social roles, characters, scenes, and stories), since other layers can be added easily with the same approaches. Therefore, we first presented computational definitions of narrative utterances on the four layers and relationships between the utterances. Then, we proposed methods for bridging three gaps between the four layers: Char2Vec, Scene2Vec, and Story2Vec-H.

In the evaluation based on the real movies, the proposed model was capable of representing various narrative utterances with a vector, which could not get interests from the existing studies. However, Story2Vec-H could not significantly outperform the existing story embedding methods (Story2Vec-F/D/U). Also, Scene2Vec and the existing scene embedding method (Scene2Role2Vec) could not exhibit reasonable accuracy commonly. The relatively low performance of the proposed model might come from mainly two reasons. First, narrative utterances have different significance. However, the proposed model treats all the narrative utterances equivalently. Second, the proposed and existing embedding methods aim to generate distributed representations. Thus, components of the vector representations have different meanings and importance for an evaluation task. Nevertheless, we also dealt with all the components equivalently. To solve these problems, we will focus on the following research directions in further research.

- Significance of Narrative Utterances: We should not deal with the climax scenes as with small talks between minor characters. Also, the protagonist will affect human perception for stories far more than extra characters. However, we consider them equally for learning representations of coarser utterances. To emphasize the significant utterances, we can conduct learning procedures multiple times for them. Dynamically adjusting learning rates can be one of the solutions. Also, just omitting insignificant utterances will be the simplest way.
- Evaluation Tasks: In this study, we assessed the quality of vector representations by estimating similarity between narrative utterances. Also, the similarity was measured by using the cosine similarity and Euclidean distance. However, each component of the vectors will represent different narrative features. As the significance of the narrative features depends on a task, the importance of the vector components is also dependent on the task. Therefore, to examine the characteristics of the story embedding models, we have to apply the vector representations on various tasks. Also, on conducting the evaluation task, we should employ methods that can consider the vector components independently (e.g., regression models or neural networks).

## Figures and Tables

**Table 1:** Major characters that appeared in ‘The Godfather’ (1972).

Notation	Name	Notation	Name
$c_{1}$	Don Vito Corleone	$c_{2}$	Michael Corleone
$c_{3}$	Sonny Corleone	$c_{4}$	Tom Hagen
$c_{5}$	Fredo	$c_{6}$	Kay Adams
$c_{7}$	Connie	$c_{8}$	Carlo
$c_{9}$	Apollonia	$c_{10}$	Johnny Fontane
$c_{11}$	Clemenza	$c_{12}$	Tessio
$c_{13}$	Sollozzo	$c_{14}$	Barzini
$c_{15}$	Bruno Tattaglia	$c_{16}$	Capt. McCluskey
$c_{17}$	Paulie Gatto	$c_{18}$	Fabrizio
$c_{19}$	Moe Greene		

**Table 2:** A list of the movies for comparing the vector representations of stories.

Title	Release Year	Genres
The Godfather	1972	Crime, Drama
Frankenstein	1994	Drama, Horror, Romance
Carrie	1976	Horror
Alien	1979	Horror, Sci-Fi
Amadeus	1984	Biography, Drama, History
Juno	2007	Comedy, Drama
Her	2013	Drama, Romance, Sci-Fi
The Bodyguard	1992	Action, Drama, Music
Rocky	1976	Drama, Sport
Scarface	1983	Crime, Drama
